# The Key Role and Mechanism of Oxidative Stress in Hypertrophic Cardiomyopathy: A Systematic Exploration Based on Multi-Omics Analysis and Experimental Validation

**DOI:** 10.3390/antiox14050557

**Published:** 2025-05-07

**Authors:** Sijie Zhang, Tianzhi Li, Shiyi Sun, Yujiao Jiang, Yuxin Sun, Yan Meng

**Affiliations:** Key Laboratory of Pathobiology, Department of Pathophysiology, Ministry of Education, College of Basical Medical Sciences, Jilin University, 126 Xinmin Street, Changchun 130021, China; sijie23@jlu.edu.cn (S.Z.); tzli23@mails.jlu.edu.cn (T.L.); sysun24@mails.jlu.edu.cn (S.S.); jiangyj22@jlu.edu.cn (Y.J.); sunyux@jlu.edu.cn (Y.S.)

**Keywords:** hypertrophic cardiomyopathy, oxidative stress, drug prediction, molecular docking

## Abstract

Hypertrophic cardiomyopathy (HCM), characterised by abnormal ventricular thickening, involves complex mechanisms including gene mutations, calcium dysregulation, mitochondrial dysfunction, and oxidative stress. Oxidative stress plays a pivotal role in the progression of HCM by mediating cardiomyocyte injury and remodelling. This study systematically analysed HCM transcriptomic data using differential gene expression, weighted gene co-expression network analysis (WGCNA), and unsupervised consensus clustering to identify key genes and classify HCM subtypes. Four oxidative stress-related characteristic genes (*DUSP1*, *CCND1*, *STAT3*, and *THBS1*) were identified using LASSO regression, SVM-RFE, and Random Forest algorithms. Their functional significance was validated by immune infiltration analysis, drug prediction using the cMAP database, and molecular docking. Single-cell RNA sequencing revealed their cell-type-specific expression, and in vitro experiments confirmed their role in HCM. These findings provide insights into oxidative stress mechanisms and potential therapeutic targets for HCM.

## 1. Introduction

Hypertrophic cardiomyopathy (HCM) is one of the most common hereditary cardiomyopathies worldwide, with a prevalence of about 1/500, and its remarkable genetic heterogeneity and clinical diversity have made it an important research topic in the field of cardiovascular disease [1]. HCM is characterised by abnormal thickening of the ventricular wall (in particular asymmetric hypertrophy of the interventricular septum), often accompanied by diastolic dysfunction, arrhythmias, heart failure, and even sudden death, and is a serious threat to the lives of patients [2]. In this process, oxidative stress, as a key driver of the pathological process in HCM, runs through the entire process of cardiomyocyte damage and remodelling, causing mitochondrial dysfunction, protein and lipid damage, myocardial fibrosis, and inflammatory responses.

Oxidative stress is both a downstream effector of gene mutations and metabolic abnormalities in HCM and a core pathogenic factor driving myocardial remodelling. In patients with HCM, mutations in genes encoding myofilament proteins lead to abnormal myofilament contractile function, which in turn leads to a decrease in mitochondrial electron transport chain efficiency and ATP production, and promotes the overproduction of ROS [3]. Excess ROS act directly on cardiomyocytes, destroying myofilament structure and function through the oxidative modification of sulfhydryl groups or the carbonylation of key contractile proteins such as actin and myosin [4]; More importantly, ROS induces myofibroblasts to differentiate into myofibroblasts by activating the TGF-β/Smad signalling pathway, promoting collagen deposition and myocardial fibrosis [5]. Therefore, intervention strategies targeting oxidative stress may break the vicious cycle of HCM and provide important new directions for the development of disease-modifying therapies.

To systematically explore the mechanism of oxidative stress in HCM, this study integrated multi-omics data analysis and experimental validation to conduct in-depth exploration. First, oxidative stress-related genes (OXs, threshold score > 7) were screened based on the GeneCards database and combined with the transcriptome data of HCM patients from the GEO database. Then, differential expression analysis (DEA) and weighted gene co-expression network analysis (WGCNA) were performed. Weighted gene co-expression network analysis (WGCNA) was performed to identify oxidative stress modules and candidate genes closely associated with HCM. A protein–protein interaction (PPI) network was then constructed to further screen the core genes, three machine learning algorithms (LASSO regression, Support Vector Machine Recursive Feature Elimination (SVM-RFE), and Random Forest) were used to identify key oxidative stress regulators, and a logistic diagnostic model was constructed to evaluate their diagnostic efficacy. Meanwhile, the correlation between these key genes and immune cell infiltration was analysed using the MCPcounter algorithm to reveal their role in the immune microenvironment of HCM. To further explore potential therapeutic targets, small-molecule regulatory drugs targeting these characteristic genes were searched using the Connectivity Map (cMAP) database, and the robust binding capability between the drugs and targets was assessed using molecular docking analysis. Finally, an in vitro model of HCM was established in H9C2 cells, and qRT-PCR experiments were performed to verify the biological functions of the key genes and their potential as therapeutic targets. This study not only deepens the understanding of the pathological mechanisms of HCM but also provides an important theoretical and experimental basis for the development of targeted intervention strategies based on oxidative stress.

## 2. Materials and Methods

### 2.1. Data Acquisition

**Figure 1:**
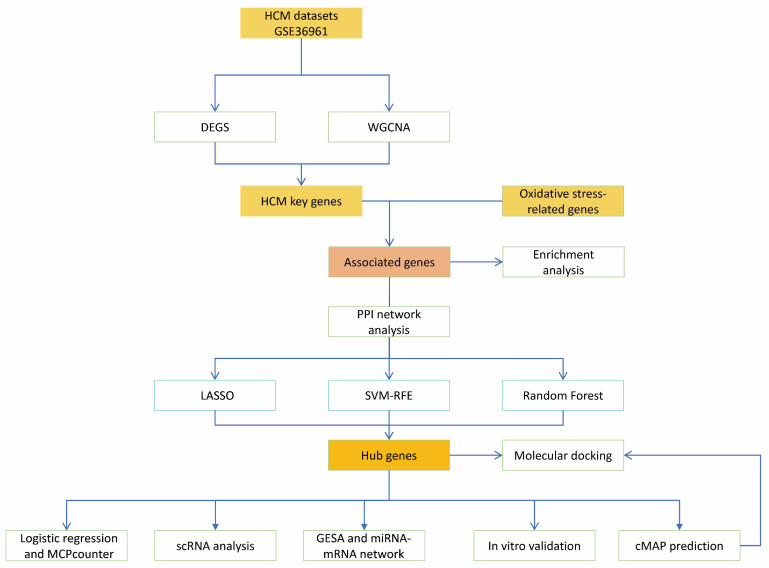
Study flow.

In this study, RNA sequencing data of HCM patients and a normal group were obtained from the Gene Expression Omnibus (GEO, https://www.ncbi.nlm.nih.gov/geo/) (accessed on 1 April 2025) [6] to ensure the reliability and breadth of the data sources. The training set data were obtained from GSE36961 (platform GPL15389), which contains a large amount of transcriptome information from HCM patients and healthy controls, providing rich genetic background data for subsequent analyses. The single-cell RNA sequencing dataset GSE137167 was also used for subsequent validation to reveal the expression characteristics of key genes in different cell types and their biological significance. To focus on oxidative stress-related genes (OXs), we retrieved 1719 OXs (threshold score > 7) from the GeneCards database (https://www.genecards.org) (accessed on 1 April 2025) [7] and calculated the gene enrichment scores for the test set using the Single-Sample Genome Enrichment Analysis (ssGSEA) algorithm in the R package gsva [8]. Changes in oxidative stress-related functions were assessed. The flow of the study is shown in Figure 1.

### 2.2. Identification and Functional Annotation of Differentially Expressed Genes (DEGs)

To systematically identify the differentially expressed genes (DEGs) between the HCM patients and healthy controls, we used the ‘limma’ R package (version 3.56.2) [9] to normalise the transcriptome data and analyse for differences. The screening criteria were set as an adjusted *p*-value < 0.05 and an absolute log2 fold change (|log2FC|) > 0.5 to ensure that the identified genes were statistically significant and biologically relevant. To further visualise the expression patterns of the DEGs, heatmaps were generated using the ‘pheatmap’ R package (version 1.0.12) and volcano maps were plotted using the ‘ggplot2’ R package (version 3.5.0) to fully reveal the expression patterns of the genes between the HCM and normal samples.

### 2.3. Weighted Gene Co-Expression Network Analysis (WGCNA)

Based on the GSE36961 dataset, the ‘WGCNA’ R package (version 1.72-5) was used to construct a weighted gene co-expression network, aiming at exploring the co-expression relationship and modularity between genes. Firstly, the top 10,000 genes with the highest variance were selected, their Pearson correlation coefficients were calculated to construct a neighbour-joining matrix, and the appropriate soft thresholding power was chosen to ensure that the network conformed to the scale-free topology. Subsequently, the neighbour-joining matrix was converted to a Topological Overlap Matrix (TOM), and gene modules with similar expression patterns were identified by the Dynamic Tree Cut method. The modules were further combined with a correlation analysis between the modules and HCM phenotypes in order to screen out the modules that were significantly associated with the disease. Finally, these module genes were intersected with the differentially expressed genes (DEGs) and oxidative stress-related genes (OXs) to identify hub genes that were highly related to HCM, which laid the foundation for our subsequent functional analysis.

### 2.4. Functional Enrichment Analysis and Protein–Protein Interaction (PPI) Network Construction

In order to deeply analyse the functional characteristics of the hub genes, we performed Gene Ontology (GO) functional enrichment analysis and Kyoto Encyclopedia of Genes and Genomes (Kyoto) functional enrichment analysis using the ‘clusterProfiler’ R package (version 4.8.3) [10]. For the Kyoto Encyclopedia of Genes and Genomes (KEGG) pathway analysis, the screening criterion was an adjusted *p*-value < 0.05 in order to ensure the reliability of the enrichment results, and the GO analysis covered three levels, namely Biological Process (BP), Molecular Function (MF) and Cellular Component (CC). The GO analysis covered the Biological Process (BP), Molecular Function (MF) and Cellular Component (CC), and comprehensively revealed the biological functions of the hub genes, while the KEGG analysis focused on their potential roles in the signalling pathways.

To further explore the interactions of hub genes, we constructed a protein–protein interaction (PPI) network based on the STRING database (https://cn.string-db.org/) (accessed on 1 April 2025), and the filtering condition was set as a confidence score > 0.7 to ensure the biological relevance of the network. Subsequently, the PPI network was imported into Cytoscape software (version 3.9.1) for visualisation and the core sub-network was filtered using the MCODE plug-in (Molecular Complex Detection) (threshold > 0.4) to focus on the key node gene.

### 2.5. Machine Learning Model Construction and Trait Gene Screening

In order to systematically screen the trait genes closely related to HCM, three classical machine learning algorithms for multi-dimensional trait screening were used in this study. First, the Least Absolute Shrinkage and Selection Operator (LASSO) algorithm was used to shrink the high-dimensional data by combining it with the ‘glmnet’ R package [11]. LASSO could effectively remove the redundant features and retain the important features by introducing an L1 regularisation term to achieve variable selection. By introducing the L1 regularisation term, LASSO could effectively remove the redundant features and retain the key genes. Secondly, a Support Vector Machine Recursive Feature Elimination (SVM-RFE) model was constructed based on the ‘e1071’ R package, and the Mean Misclassification Error (MME) was evaluated by 5-fold cross-validation. The low-contributing features were gradually eliminated until the model performance was optimal [12]. Finally, a Random Forest (RF) model was constructed using the ‘randomForest’ R package, the importance of each feature was evaluated by calculating the Gini index, and the genes with the highest contribution to classification were filtered out [13]. The overlapping biomarkers identified by the three algorithms were defined as characteristic OXs to ensure the stability and reliability of the results.

### 2.6. Construction and Validation of Diagnostic Model

To further evaluate the diagnostic efficacy of characteristic genes in HCM, we constructed a diagnostic nomogram model based on the ‘rms’ R package. The model integrated the expression levels of multiple characteristic genes, quantified the contribution of each gene to the disease risk by assigning weights, and plotted the corresponding calibration curves to evaluate the prediction accuracy of the model [14]. In addition, the ‘pROC’ R package (version 1.18.0) was used to construct the Receiver Operating Characteristic (ROC) curve and calculate the Area Under the Curve (AUC) [15]. The closer the AUC value was to 1, the better the classification performance of the model. The performance of the characterised genes under different sensitivity and specificity conditions was analysed comprehensively to assess their potential as HCM diagnostic markers.

### 2.7. Correlation Analysis Between Characteristic Genes and Immune Cells

To analyse the immune microenvironment associated with HCM and its correlation with characteristic genes, the ‘MCPcounter’ R package (version 1.2.0) [16] was used to quantify the level of immune cell infiltration. By comparing the relative abundance of different immune cell types in HCM patient samples, the dynamic changes in immune cell populations during disease progression were revealed. Pearson correlation coefficients were also used to assess the correlation between characteristic genes and immune cell infiltration levels and to explore the potential mechanisms of their role in regulating inflammatory responses, fibrosis, and myocardial remodelling. This analysis not only revealed the functional status of immune cells in the pathophysiological process of HCM but also provided a theoretical basis for the development of immune-targeted therapeutic strategies.

### 2.8. Hub Gene Enrichment and miRNA-mRNA Regulatory Network Analysis

To explore the potential regulatory mechanisms of hub genes in HCM and their association with disease states, Single-Sample Gene Set Enrichment Analysis (GSEA) was performed on the screened characteristic genes in this study. This analysis aimed to reveal the functional distribution of the hub genes in specific signalling pathways and biological processes and their possible mechanisms of action. In addition, to further resolve the post-transcriptional regulatory networks of the key genes, miRNA-mRNA interactions were constructed using the NetworkAnalyst platform (https://www.networkanalyst.ca/) (accessed on 1 April 2025) [17], and miRNAs significantly associated with them were screened. The miRNA-mRNA regulatory networks were then imported into Cytoscape software for visualisation and identified using topological parameters (e.g., degree, betweenness centrality) to identify core nodes and reveal potential regulatory hubs. This multi-level analysis strategy provided important clues to understanding the complex network of gene regulation in HCM.

### 2.9. Drug Prediction and Molecular Docking Analysis

In order to explore potential therapeutic compounds and reveal their mechanisms of action, we conducted drug prediction analyses based on the Connectivity Map (CMAP) (https://clue.io/) (accessed on 2 April 2025.) database, which contains 6100 instances of 1309 small-molecule drugs, each of which is accompanied by information on the expression profiles of a specific drug and its corresponding genes [18]. By uploading the differential expression profiles of the pivotal genes into the CMAP database, we predicted the small-molecule compounds that might be used in the treatment of HCM and screened drug candidates with significant negative correlations. To further validate the binding ability between the predicted compounds and the encoded proteins of the featured genes, we performed molecular docking (MD) analysis. The specific steps were as follows: Firstly, the 3D structure files of the core target proteins were obtained from the UniProt database; then, the structure files of the drug candidates in SDF format were downloaded from the PubChem database and converted to mol2 format using OpenBabel software (Version-2.3.2) in order to meet the requirements of the subsequent analyses. Next, AutoDock software (Version-4.2.6) was used to simulate the molecular docking between the protein receptor and the small-molecule ligand and calculate the Binding Free Energy (BFE) of the two. The lower the Binding Free Energy value, the stronger the binding affinity between the small molecule and the target protein. Finally, PyMOL software (Version-3.1) was used to visualise the molecular docking results in order to show the interaction patterns between the small molecules and the target proteins, including key interaction sites such as hydrogen bonds, hydrophobic interactions, and π-π stacking [19]. This analysis Thank you. I’ve changed. provided an important experimental basis for the development of targeted therapeutic drugs against HCM.

### 2.10. Analysis of Single-Cell RNA Sequencing Data (scRNA-seq)

The single-cell RNA sequencing (scRNA-seq) data of GSE137167 were normalised and downstream analysed using the ‘Seurat’ package (version 4.3.0) in the R software (R-4.2.2). The quality control steps were as follows: first, low-quality cells, including cells expressing less than 300 or more than 2500 genes and genes expressed in fewer than 3 cells, were excluded to remove empty droplets or potential duplexes and multiplexes; second, cells with more than 10% mitochondrial RNA were considered to be of poor quality and filtered out. To normalise the data, the gene expression levels of the remaining cells were multiplied by 10,000 and log-transformed. Subsequently, Highly Variable Genes (HVGs) were filtered based on the standard deviation of gene expression and used to capture heterogeneity between cells. To scale the data, the gene expression matrix was normalised using the ScaleData function in the Seurat package, with the mean expression of each gene between cells set to 0 and the variance set to 1. Next, principal component analysis (PCA) was performed on the top 3000 Highly Variable Genes, and 13 major principal components were screened for use in our subsequent analysis. The cells were visualised in two dimensions by the UMAP algorithm, and the GPT4celltype tool was used to annotate the cell types and perform the associated visualisation analysis [20]. In addition, in order to infer and analyse the intercellular communication network, we performed intercellular communication interactions analysis using the CellChat package (v1.6.1) [21] to reveal the signalling and functional associations between different cell types, which provided important support for resolving the intercellular regulatory network in HCM.

### 2.11. Cell Culture and In Vitro Model Construction

Cells in the H9c2 cell line, widely used in cardiovascular disease research, were purchased from Procell Life Science and Technology Co. The cells were cultured in complete DMEM containing 1 g/L glucose and 10% foetal bovine serum (FBS) in a constant temperature incubator at 37 °C with 5% CO_2_. The experimental design was as follows: H9c2 cells in the logarithmic growth phase (density of approximately 1 × 10^5^ cells/mL) were harvested and inoculated into 35 mm cell culture dishes, with approximately 1 × 10^5^ cells per dish. Twenty-four hours later, the cells were randomly divided into two groups: the control group (control group) and the AngII stimulation group (AngII group). The control group received 2 mL of DMEM containing 1% FBS for 48 h, while the AngII group received 0.1 μmol/L angiotensin II (AngII) dissolved in 2 mL of DMEM containing 1% FBS, also for 48 h. The medium was changed every 24 h in both groups to ensure the consistency of the experimental conditions. This in vitro model simulated the pathological conditions of HCM and provided an experimental basis for validating the functions of the characterised genes and their potential as therapeutic targets.

### 2.12. qPCR In Vivo Validation

RT-PCR was performed to detect the mRNA levels of the four characterised genes. Total RNA was extracted from cells using TRIzol reagent according to the manufacturer’s instructions and used for cDNA synthesis and RT-PCR; primer sequences are listed in Table 1. The RT-qPCR data were analysed using the ΔΔCT method, and GAPDH was used as an internal control to normalise the expression of all the genes.

### 2.13. Statistical Analysis

All data are expressed as mean ± standard deviation (SD). GraphPad Prism 9.0 was used for statistical analysis and graphing. One-way ANOVA and Dunnett’s post hoc tests were used to compare differences between multiple groups, and *t*-tests were used to compare differences between two groups. *p* < 0.05 was considered statistically significant.

## 3. Results

### 3.1. Characterisation of HCM Based on Oxidative Stress-Related Genes

**Figure 2:**
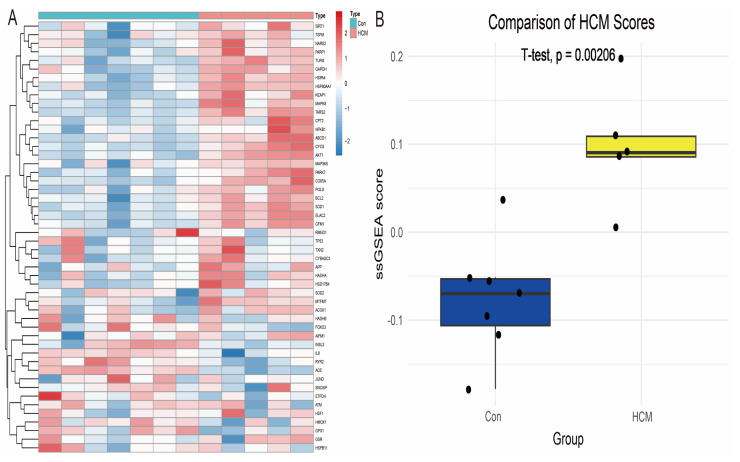
The analysis of the differences in oxidative stress scores in HCM. (**A**) A heatmap display of the expression profiles of the oxidative stress-related genes in the GSE68316 dataset, with the colour shades reflecting the level of gene expression. (**B**) A comparison of oxidative stress scores between the patient group and the healthy control group, with the scores in the patient group being significantly elevated, suggesting the potential driving role of oxidative stress in HCM.

In order to systematically develop gene signatures that could distinguish patients with hypertrophic cardiomyopathy (HCM), we extracted 1719 genes related to oxidative stress (OS) from the GeneCards database and performed Single-Sample Gene Set Enrichment Analysis (ssGSEA) based on these genes in the GSE68316 dataset. The oxidative stress-related scores of each sample were calculated by the ssGSEA algorithm (Figure 2A). Further statistical analysis showed that the oxidative stress score in the patient group was significantly higher than that in the healthy control group (*p* = 0.047, Figure 2B), suggesting the critical role of oxidative stress in HCM.

### 3.2. Identification of Differentially Expressed Genes (DEGs) in GSE36961 Cohort

**Figure 3:**
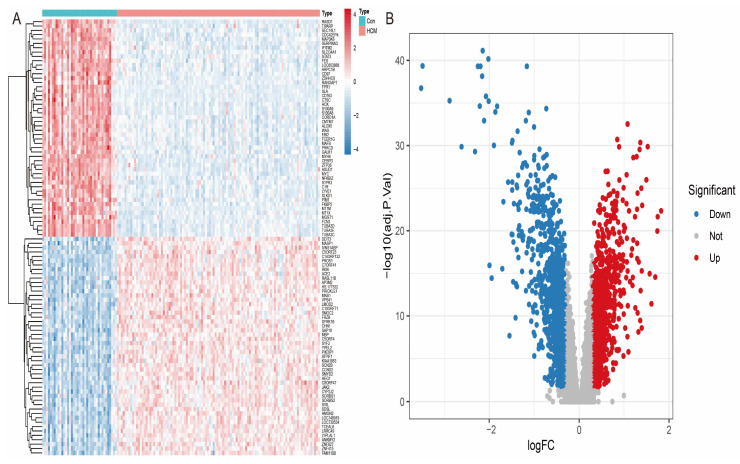
Visual analysis of differentially expressed genes (**A**) Heatmap showing cluster analysis of differentially expressed genes: rows represent genes, columns represent samples, and colour gradient reflects gene expression level. (**B**) Volcano plot showing distribution of differentially expressed genes: red dots represent up-regulated genes, blue dots represent down-regulated genes, and grey dots represent genes with no significant difference.

To systematically identify the differentially expressed genes (DEGs) between HCM patients and healthy individuals, we performed differential expression analysis on the GSE36961 dataset. The screening criteria were set as an adjusted *p*-value < 0.05 and absolute log2 fold change (|log2FC|) > 0.5. The results showed that a total of 888 genes showed significant differential expression, including 306 up-regulated genes and 502 down-regulated genes. To further visualise the differential expression patterns, we used the ‘pheatmap’ R package to generate heatmaps (Figure 3A) and the ‘ggplot2’ R package to draw volcano maps (Figure 3B). The heatmap clearly shows the gene expression differences between the HCM patients and healthy controls, while the volcano plot highlights the significantly up- and down-regulated genes.

### 3.3. WGCNA Identifies Modular Genes Associated with HCM

**Figure 4:**
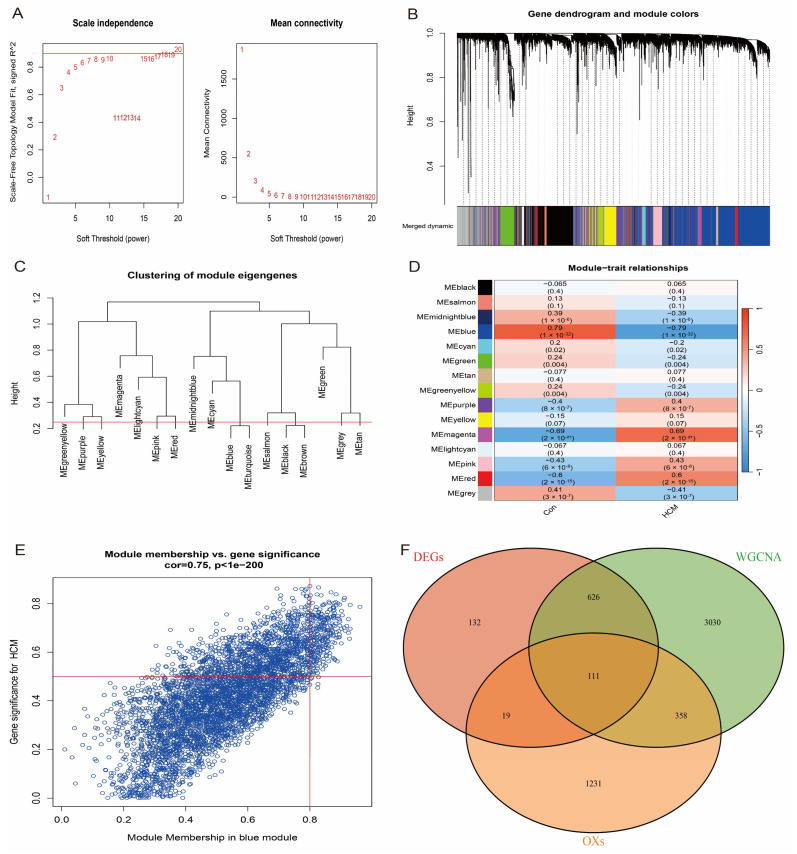
WGCNA identifies key modules of HCM. (**A**) Scale-free fit indices and average connectivity analysis for different soft-threshold powers; β = 8 chosen to ensure that network met scale-free properties. (**B**) Hierarchical clustering dendrogram showing all genes classified into 15 modules, with different colours representing different modules. (**C**) Cluster dendrogram of module-characterised genes reflecting inter-module correlation. (**D**) Heatmap of module–feature correlation, where first row for each cell row is correlation coefficient and second row is *p*-value. (**E**) Scatter plot of blue module showing correlation between genes within module and HCM phenotypes. (**F**) Venn diagram showing intersection of differentially expressed genes, oxidative stress-related genes, and genes of blue module. Finally, 111 candidate genes were identified.

To further identify the hub genes most closely associated with HCM, we performed weighted gene co-expression network analysis (WGCNA) on the HCM patients and healthy individuals in the GSE36961 dataset. First, the top 10,000 genes with the highest variance were selected to construct the gene co-expression network, and the network was ensured to be close to a scale-free distribution by a soft-threshold power parameter β = 8 (scale-free topology, Figure 4A). Subsequently, gene modules associated with clinical features were detected based on the Topological Overlap Matrix (TOM). The analysis showed that the blue module had the highest correlation with HCM (correlation coefficient = 0.79, *p*-value = 1 × 10^−32^, Figure 4B–D). From the blue module (Figure 4E), 4124 genes with module correlation coefficients above 0.5 were screened and further intersected with differentially expressed genes (DEGs) and oxidative stress-related genes, and 111 candidate genes were finally identified (Figure 4F). These genes were considered to be hub genes closely associated with HCM.

### 3.4. Enrichment Analysis and PPI Network

**Figure 5:**
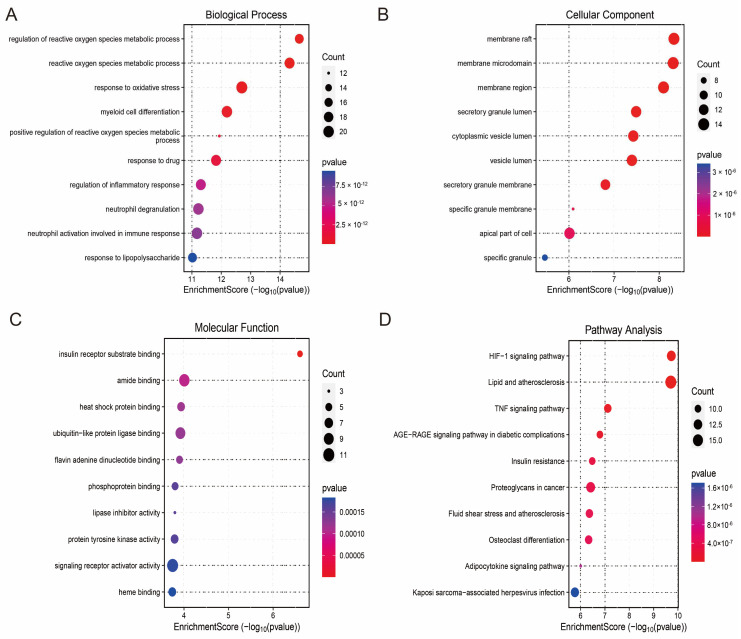

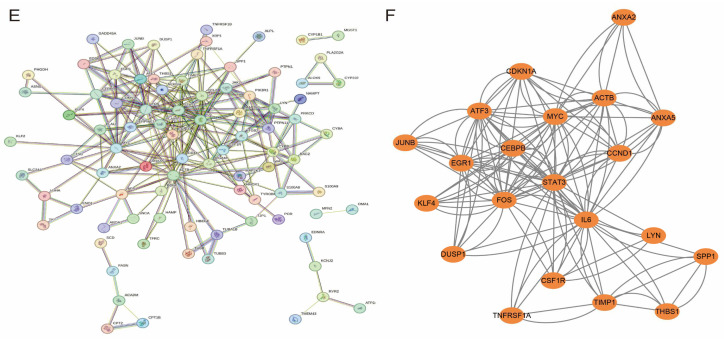
Functional enrichment analysis of hub genes and PPI network construction. (**A**) Biological Process (BP) enrichment analysis showing major biological events involved in hub genes; (**B**) Cell Component (CC) enrichment analysis revealing subcellular localisation of hub genes; (**C**) Molecular Function (MF) enrichment analysis showing functional characteristics of hub genes; (**D**) KEGG pathway enrichment analysis showing major signalling pathways involved in hub genes; (**E**) protein–protein interaction (PPI) network graph, with node size reflecting importance of genes; (**F**) core sub-network of MCODE screen containing 21 candidate hub genes.

To explore the biological functions of the hub genes and their related pathways in HCM, we performed Gene Ontology (GO) and Kyoto Encyclopedia of Genes and Genomes (KEGG) enrichment analyses. The results of the GO functional enrichment analysis showed that the hub genes were significantly involved in neutrophil activation at the Biological Process (BP) level. At the Biological Process (BP) level, the hub genes were significantly involved in key immune and metabolic events such as neutrophil activation, inflammatory response regulation, reactive oxygen species (ROS) metabolism regulation, and oxidative stress response. At the Cellular Component (CC) level, they were mainly enriched in subcellular structures such as the vesicle lumen, membrane region, and top of the cell; at the Molecular Function (MF) level, the hub genes exhibited functional characteristics such as signalling receptor activator activity, heat shock protein binding capacity, and lipase inhibitor activity (Figure 5A–C). KEGG pathway enrichment analysis further revealed that the hub genes were associated with fluid shear-stress-mediated atherosclerosis, TNF signalling, the HIF-1 signalling pathway, and the HIF-1 signalling pathway. TNF signalling, HIF-1 signalling, and adipocytokine signalling were closely associated with fluid shear-stress-mediated atherosclerosis (Figure 5D). These results suggest that the hub genes not only play a central role in the regulation of cellular energy metabolism and responses to oxidative stress in HCM but may also be involved in the disease process through immune-related mechanisms.

To further identify the key hub genes, we constructed a protein–protein interaction (PPI) network based on the STRING database and set the confidence level to 0.7 (Figure 5E). The PPI network was then imported into Cytoscape software for visualisation, the network modules were screened using the MCODE plug-in (scoring threshold > 0.4), and 21 candidate hub genes were finally identified (Figure 5F).

### 3.5. Feature Gene Selection

**Figure 6:**
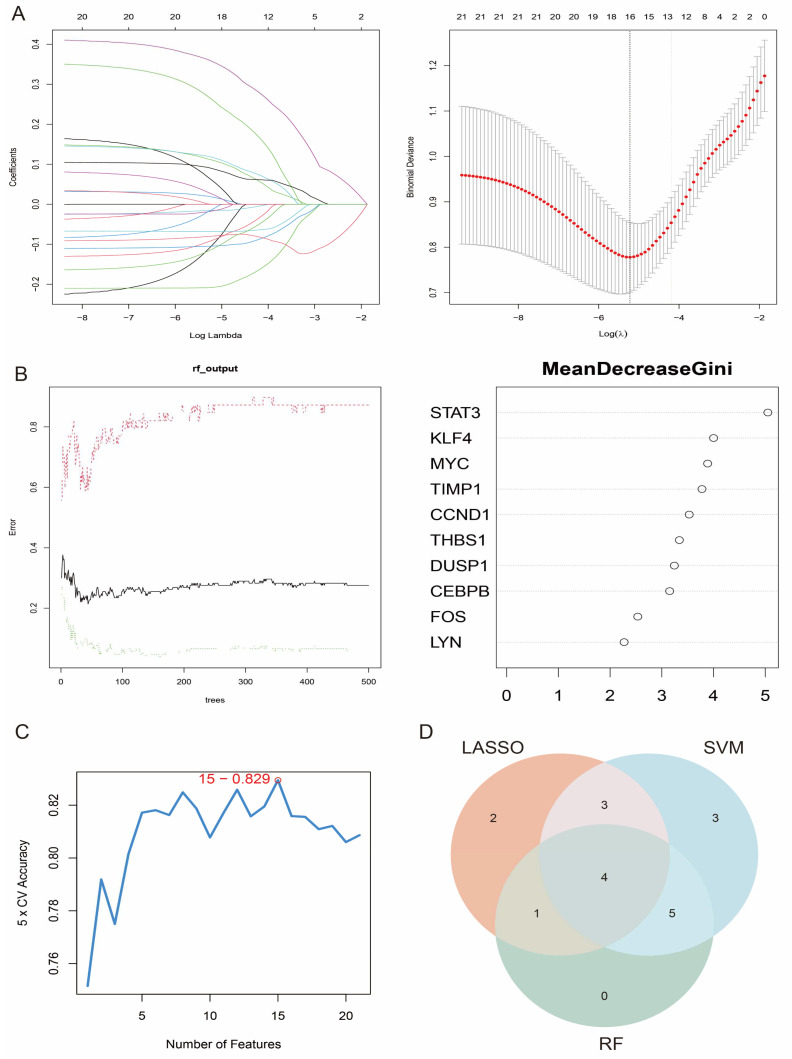
Feature gene selection and validation. (**A**) Coefficient path diagram of LASSO regression analysis showing feature gene selection process; (**B**) importance score ranking of feature genes in Random Forest model; (**C**) 15 genes flagged by SVM-RFE method and their classification accuracies; (**D**) Venn diagrams of screening results of three algorithms, which finally identified four core genes.

To further screen the most important feature genes from the 21 candidate genes, three commonly used feature selection algorithms were used: the Support Vector Machine Recursive Feature Elimination (SVM-RFE), the Random Forest (RF), and the Least Absolute Shrinkage and Selection Operator (LASSO) algorithms. Ten genes were screened by LASSO regression analysis (Figure 6A), and their coefficient path diagrams showed the stability of the featured genes in the regularisation process. The 21 candidate genes were then ranked by importance scores using the Random Forest approach, and the top 10 feature genes were selected for further analysis (Figure 6B). In addition, the SVM-RFE method labelled 18 genes with a classification accuracy of 0.829 (Figure 6C). Finally, through the intersection analysis of the screening results of the three algorithms, we identified four core genes (*DUSP1*, *THBS1*, *STAT3*, and *CCND1*), which were identified as the key feature genes of oxidative stress in HCM (Figure 6D).

### 3.6. Evaluation of Characteristic Genes

**Figure 7:**
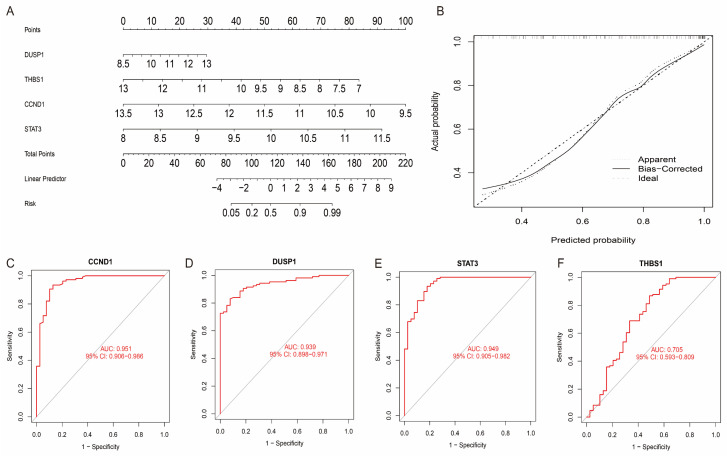
Construction and evaluation of HCM diagnostic models. (**A**) Column-line diagram model for predicting HCM risk, showing weighting of each signature gene on disease risk. (**B**) Calibration curves of column-line diagram model, showing degree of fit between predicted probabilities and actual observed values. (**C**–**F**) ROCs of four characteristic genes and their corresponding AUC values, indicating their high accuracy in predicting clinical outcome.

To evaluate the accuracy of these four characteristic genes in diagnosing the disease, we constructed a diagnostic model by integrating these genes using logistic regression in the GSE36961 dataset. The performance of the model was evaluated using the Receiver Operating Characteristic (ROC) curve and its Area Under the Curve (AUC). The results showed that the four characteristic genes had excellent diagnostic potential in discriminating HCM patients from healthy controls, with AUC values greater than 0.7 (Figure 7A–F). This result indicates that the diagnostic model constructed based on the characteristic genes has a high predictive performance and can effectively identify HCM patients. To further optimise the clinical applicability of the model, we constructed a diagnostic nomogram based on the ‘rms’ R package and evaluated the predictive accuracy of the model using calibration curves. The nomogram model combines the expression levels of the four characteristic genes to visualise the contribution of each factor to the risk of HCM.

### 3.7. Correlation Between Characterised Genes and Immune Cells

**Figure 8:**
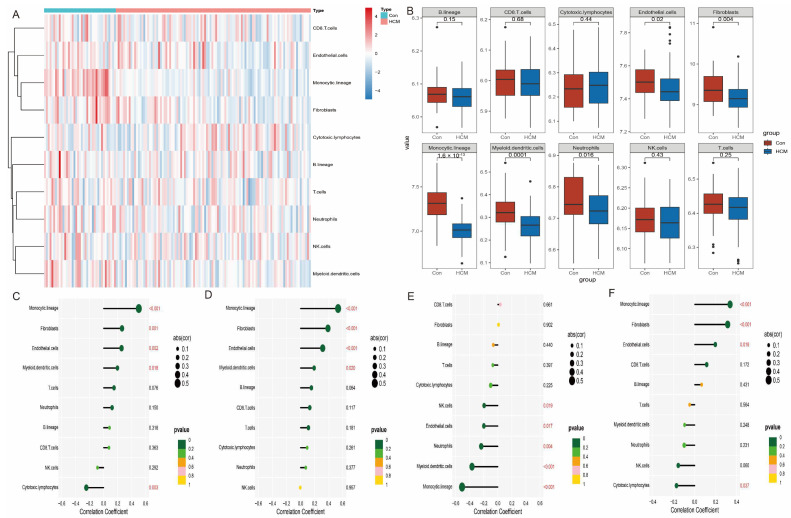
Distribution of immune cells in HCM and correlation of characteristic genes. (**A**) Heatmap showing degree of immune cell infiltration, with colour shades reflecting cell abundance. (**B**) Differential analysis of immune cell abundance between HCM group and control group. (**C**–**F**) Heatmap of correlation of *CCND1*, *DUSP1*, *STAT3*, and *THBS1* with 10 types of immune cells to reveal potential association of characteristic genes with immune microenvironment.

We performed a systematic analysis of immune cell infiltration in the HCM group compared to the normal control group using the MCPcounter algorithm (Figure 8A,B). The results showed that the abundance of several immune-related cell types was significantly lower in the HCM group compared to the normal control group, including endothelial cells, fibroblasts, monocytes, myeloid dendritic cells, and neutrophils. Further correlation analysis showed that the expression levels of the four characteristic genes were significantly correlated with most immune cell types (Figure 8C–F). For example, the expression levels of *CCND1* and *STAT3* were negatively correlated with the infiltration of endothelial cells and fibroblasts, while *DUSP1* and *THBS1* were closely associated with the distribution of monocytes and myeloid dendritic cells. This result suggests that the characterised genes may play an important role in the development and progression of HCM by regulating the function of immune cells or their distribution in tissues.

### 3.8. Characteristic Gene Enrichment Analysis and miRNA-mRNA Regulatory Network

**Figure 9:**
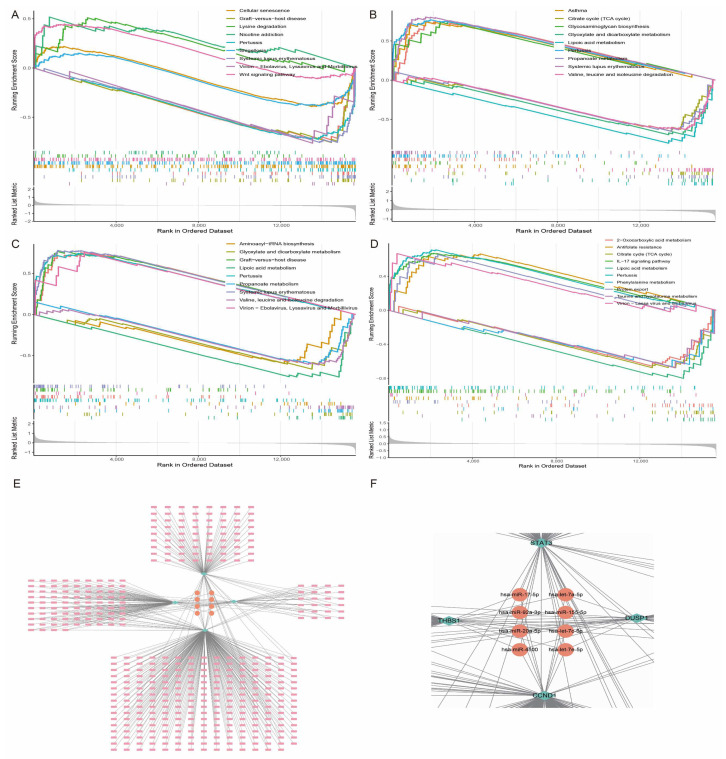
Functional enrichment analysis and miRNA-mRNA regulatory network of trait genes. (**A**–**D**) GSEA of *CCND1*, *DUSP1*, *STAT3*, and *THBS1*, showing their enrichment in key pathways. (**E**) miRNA-mRNA regulatory network diagram of four trait genes, where node size reflects strength of regulatory relationship. (**F**) is an enlargement of the middle of the E plot, the core miRNA for hub gene interactions.

Using Gene Set Enrichment Analysis (GSEA), we found that the pathways enriched by these characteristic genes were mainly involved in key biological processes such as IL-17 signalling, cellular senescence, graft-versus-host disease, and amino acid metabolism (Figure 9A–D). These results further support the central regulatory role of the above-mentioned characteristic genes in the pathogenesis of HCM. To further reveal the overall regulatory network of the feature genes and their potential molecular mechanisms, we predicted the regulatory miRNAs that these genes might bind using the NetworkAnalyst database. The analysis results showed that a variety of miRNAs have potential regulatory relationships with these feature genes, among which miR-17-5p, miR-155-5p, and miR-20a-5p are the most common target miRNAs of the four feature genes (Figure 9E,F). It is worth noting that these miRNAs have been shown to play an important role in the onset and development of HCM, which not only confirms the reliability of the results of the present study, but also provides an important theoretical basis for the subsequent in-depth discussion of the functions of the trait genes.

### 3.9. Single-Cell Analysis

**Figure 10:**
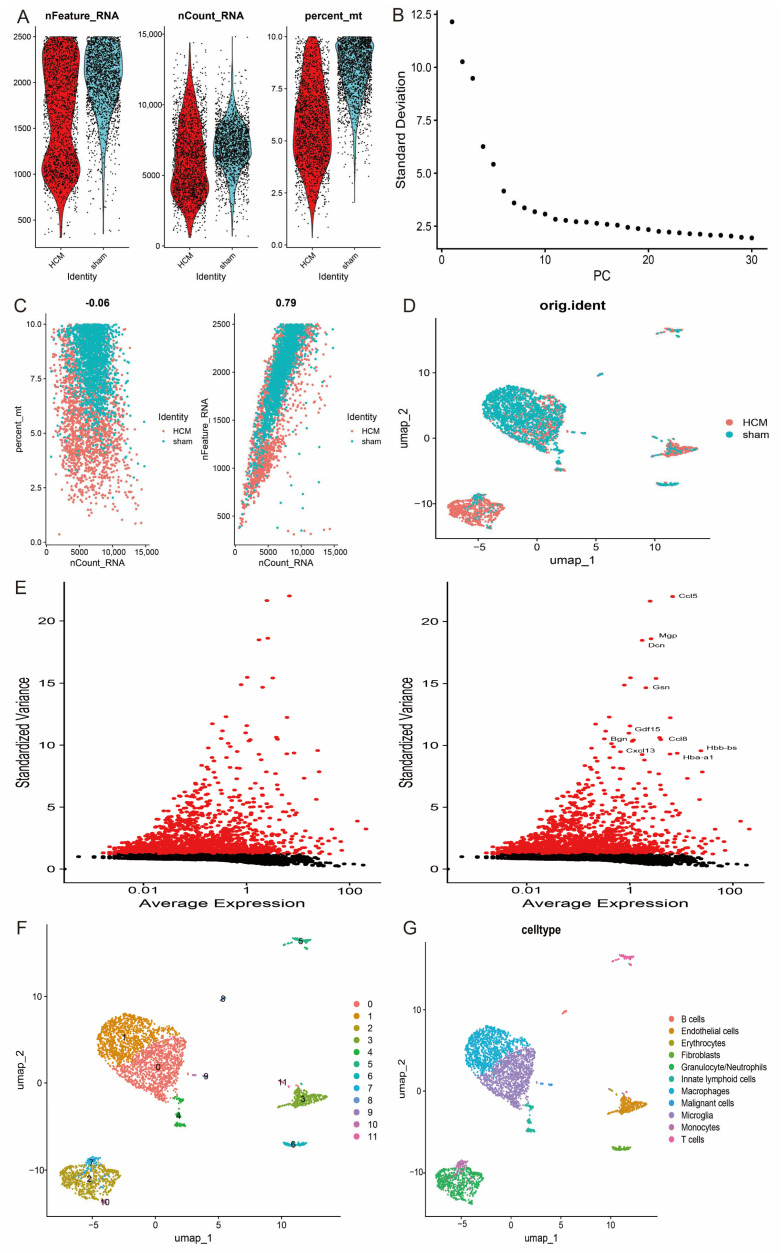
Single-cell analysis of HCM. (**A**) Quality control metrics for single-cell data, including cell counts, gene counts, and sequencing depth for each sample; (**B**) variance ranking plot of principal component analysis, demonstrating contribution of each principal component; (**C**) correlation analyses of sequencing depth with mitochondrial content and gene counts; (**D**) variance plots of genes with significant intercellular differences; (**E**) top 10 genes with highest expression variability; (**F**) 12 cell clusters revealed by UMAP downscaling analysis; (**G**) annotation results of 11 major cell types.

After extracting single-cell transcriptome data from the GSE137167 dataset, the samples were first rigorously screened based on quality control parameters. Specifically, low-quality cells were excluded by setting a threshold range (200 < nFeature_RNA < 2500 and percent.mt < 10) (Figure 10A–D). Subsequently, the standard deviation of gene expression was calculated and the top 10 genes with the highest expression variability were shown (Figure 10E). The quality-controlled single-cell data were further subjected to normalisation, log-homogenisation, and principal component analysis (PCA) to reduce technical noise and batch effects. On this basis, the data were batch corrected and integrated using the Harmony algorithm to eliminate potential experimental batch differences. To reveal intercellular heterogeneity, the data were projected into UMAP space for nonlinear downscaling and visualisation analysis, resulting in the identification of 12 distinct cell subpopulations. These subpopulations were categorised into 11 major cell types, including B cells, endothelial cells, erythrocytes, fibroblasts, granulocytes/neutrophils, lymphoid cells, macrophages, malignant cells, microglial cells, monocytes, and T cells, by functional annotation with the GPT4celltype toolkit (Figure 10F,G).

**Figure 11:**
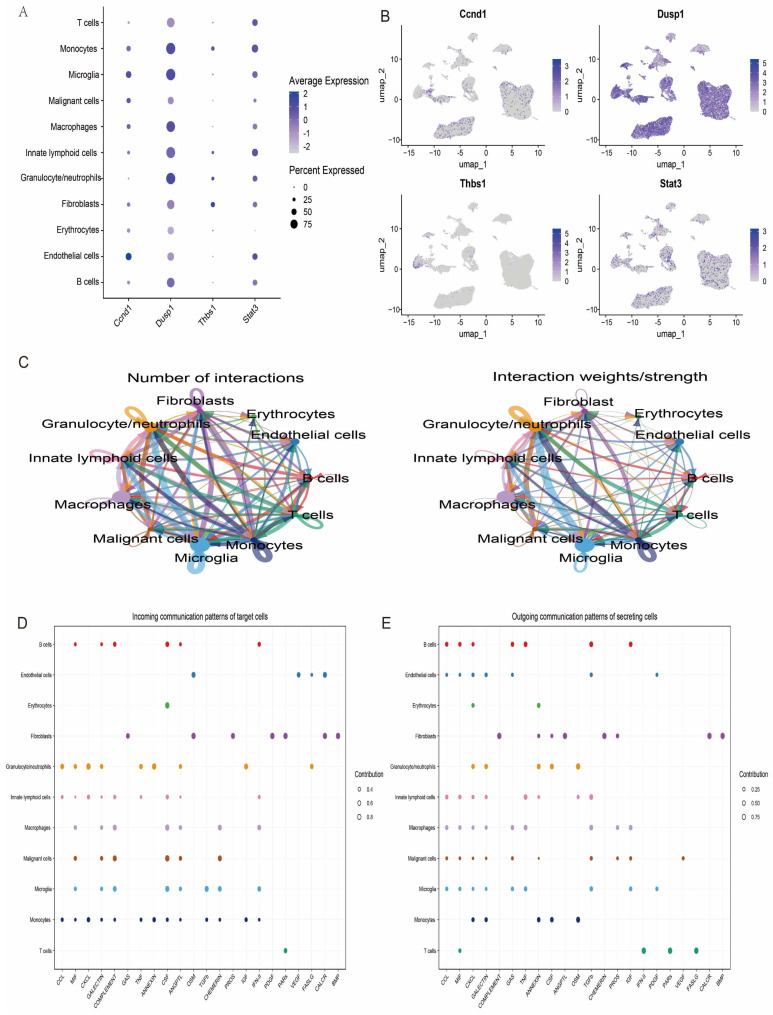
The analysis of intercellular communication between different cell types. (**A**) The ability of different cells to communicate with each other, where the size of the circle indicates the number of cells and the direction of the arrow indicates the source and reception of signals. (**B**) Bubble diagrams showing the intercellular communication signalling pathways in different cells. (**C**–**E**) The ligand–receptor pairs and cofactor-mediated signalling patterns.

Further in-depth analyses of the expression levels of the featured genes in the single-cell data were performed, and the expression patterns of the four key genes were visualised. The results showed that these genes exhibited different levels of expression activity in each of the 11 cell types (Figure 11A,B). This finding provides important clues to further elucidate the functional roles of these genes in different cell subpopulations. In addition, to explore the potential interactions between cell types, we performed a systematic analysis of the intercellular communication networks using the R package CellChat, which simulates and predicts the signalling patterns between different cell types by integrating the interaction information of ligand–receptor pairs and their cofactors. Based on this analysis, we revealed a complex communication network between multiple cell types, involving the direct interaction of ligands with receptors and the regulatory mechanisms of cofactors on signalling (Figure 11C–E).

### 3.10. Drug Prediction and Molecular Docking

**Figure 12:**
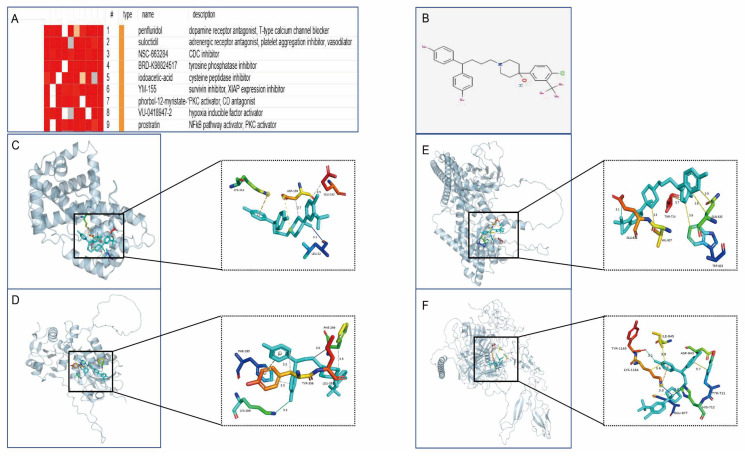
Drug prediction and molecular docking analysis. (**A**) Potential small-molecule compounds targeting characterised genes predicted from cMAP database. (**B**) 2D structure of penfluridol. (**C**–**F**) Optimal binding conformations of CCND1, DUSP1, STAT3, and THBS1 with penfluridol, showing key interaction sites such as hydrogen bonding, hydrophobic forces, and salt bridges.

To develop targeted drugs against the characterised genes, we submitted the candidate genes to the Connectivity Map (cMAP) database and successfully predicted potential therapeutic small molecules. The highest scoring compound, penfluridol, was finally selected for molecular docking analysis. The molecular docking simulation of the proteins encoded by the featured genes with penfluridol was performed using AutoDock software, and the Binding Free Energy (BFE) of both was calculated. PyMOL software was used to visualise the molecular docking results, which showed various intermolecular interactions such as hydrogen bonding, hydrophobic forces, and salt bridges (Figure 12).

### 3.11. Angiotensin-Induced mRNA Expression of Hypertrophy-Characterising Genes in H9c2 Cardiomyocytes

**Figure 13:**
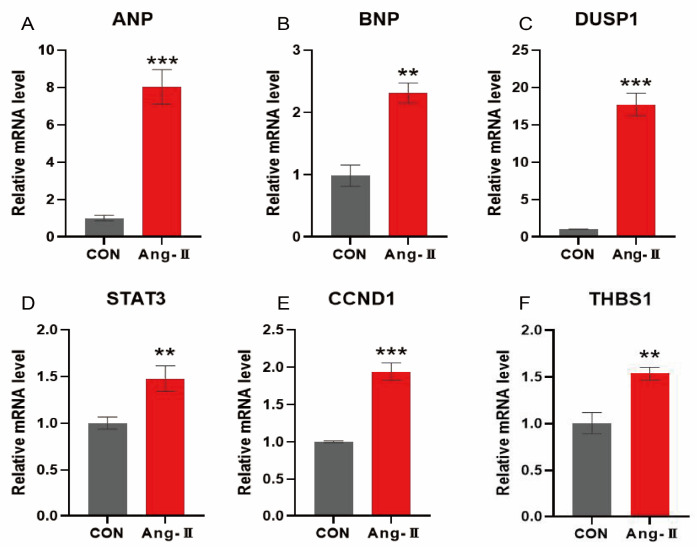
mRNA expression of genes characteristic of AngII-induced cardiomyocyte hypertrophy in H9c2 cardiomyocytes. (**A**,**B**) mRNA expression levels of ANP and BNP, showing AngII-induced cardiomyocyte hypertrophy model successfully established; (**C**–**F**) mRNA expression levels of *DUSP1*, *STAT3*, *CCND1*, and *THBS1*, showing significant changes in genes characteristic of HCM. **: *p* < 0.01, ***: *p* < 0.001.

To test the functional significance of oxidative stress characteristic genes in HCM, we established an angiotensin II (AngII)-induced hypertrophy model in H9c2 cardiomyocytes. Compared with the control group, the mRNA expression of atrial natriuretic peptide (ANP) and brain natriuretic peptide (BNP) was significantly increased in the AngII (0.1 μmol/L) group (*p* < 0.01), suggesting that the cardiac hypertrophy model was successfully established (Figure 13A,B). In addition, the mRNA expressions of CCND1, DUSP1, STAT3, and THBS1 in HCM were significantly higher than in the control group (*p* < 0.01), which further confirmed the key roles of these characteristic genes in HCM. This lays the foundation for the subsequent transformation experiments.

## 4. Discussion

Hypertrophic cardiomyopathy (HCM) is an inherited cardiomyopathy characterised by abnormal thickening of the ventricular wall. Its pathogenesis is complex and varied, involving a variety of biological processes such as gene mutations, calcium dysregulation, mitochondrial dysfunction, and oxidative stress [22,23]. Although HCM is usually not associated with a significant increase in cardiac load (e.g., hypertension or valvular disease), its clinical presentation varies widely, ranging from asymptomatic to severe heart failure and even sudden death. As one of the most common inherited heart diseases, the pathogenesis of HCM is not fully understood. Recent studies have shown that oxidative stress plays a crucial role in the development of HCM [24]. Oxidative stress refers to cell and tissue damage caused by an imbalance between the production and scavenging of reactive oxygen species (ROS). In HCM, elevated levels of ROS not only directly damage cardiomyocyte structure and function, but also participate in pathophysiological processes such as myocardial fibrosis, apoptosis, inflammatory response, and myocardial remodelling through various molecular pathways [25,26]. For example, ROS can induce cardiomyocyte hypertrophy by activating various signalling pathways (e.g., MAPK, NF-κB, and JAK/STAT pathways) and further exacerbate myocardial injury by impairing mitochondrial function and calcium homeostasis [27]. In addition, oxidative stress may regulate gene expression through epigenetic modifications (e.g., DNA methylation and histone modification) and thereby influence disease progression.

To gain insight into the specific role of oxidative stress in HCM and its potential therapeutic targets, this study adopted a multi-dimensional and systematic research strategy. First, based on the transcriptomic data of HCM patients, we performed comprehensive bioinformatics analyses, including differential expression analysis (DEG) and weighted gene co-expression network analysis (WGCNA), to identify hub genes closely related to oxidative stress. Functional enrichment analyses showed that these hub genes were significantly involved in key biological processes in the pathological mechanisms of HCM, including redox homeostasis, inflammatory response, and apoptosis. This finding is highly consistent with the central role of oxidative stress in myocardial injury previously reported in the literature. To further narrow down the candidate genes, we integrated various machine learning algorithms (e.g., Least Absolute Shrinkage and Selection Operator (LASSO) regression, Support Vector Machine Recursive Feature Elimination (SVM-RFE), and Random Forest) to perform a multi-dimensional screening of the key genes, and finally identified four characteristic genes that are closely related to oxidative stress: the results showed that these characterising genes have a high predictive value and can effectively discriminate HCM patients from healthy controls. Meanwhile, we evaluated the correlation between these characteristic genes and immune cell infiltration using the MCPcounter method and found that their expression levels were significantly correlated with the infiltration levels of several immune cell types (e.g., macrophages, T cells, and monocytes).

Gene Set Enrichment Analysis (GSEA) showed that these four characteristic genes were significantly enriched in various biological processes, such as IL-17 signalling pathway, graft-versus-host disease, and amino acid metabolism, indicating that oxidative stress plays a complex and multi-layered role in the pathogenesis of HCM. pathogenesis, suggesting that oxidative stress plays a complex and multi-layered regulatory role in the pathogenesis of HCM. In addition, miRNA-mRNA regulatory network analysis revealed that miRNAs shared by these characteristic genes (e.g., miR-17-5p and miR-155-5p) play important roles in the pathogenesis of HCM [27]. These miRNAs have been shown to be closely associated with the development of HCM, and this result further validates the reliability of our findings.

To explore the expression patterns of these characteristic genes in different cell types and their functional significance, we analysed their expression abundance in cardiomyocytes, fibroblasts, endothelial cells, and immune cells using single-cell transcriptome sequencing data. The results showed that these genes exhibited significant heterogeneous expression in different cell types, suggesting that they may exert specific biological functions in specific cell types. Combined with an analysis of intercellular communication networks using CellChat tools, we revealed complex signalling mechanisms mediated by ligand–receptor pairs and their cofactors. For example, *THBS1* promotes myocardial fibrosis by activating the TGF-β signalling pathway, whereas *STAT3* affects cardiomyocyte proliferation and apoptosis by regulating cytokine signalling.

By integrating an analysis of the Connectivity Map (CMap) database, we predicted potential therapeutic agents highly associated with the hub genes (connectivity score > 0.7). Based on the expression characteristics of the hub genes and their key roles in HCM, we screened penfluridol as a potential therapeutic candidate. While pentafluridine, as a classical antipsychotic drug, exerts its therapeutic effects mainly through antagonising dopamine D2 receptors, it has been shown to inhibit the overproduction of ROS and enhance cellular antioxidant capacity by activating antioxidant signalling pathways (e.g., Nrf2 pathway) [28]. This property may help to attenuate the myocardial remodelling and dysfunction triggered by oxidative stress in HCM. Meanwhile, its anti-inflammatory potential has been demonstrated in several studies [29], and it may exert a protective effect by inhibiting the expression of pro-inflammatory factors (e.g., IL-6 and TNF-α) as well as modulating inflammation-related signalling pathways, such as MAPK and NF-κB, and, thus, may have potential therapeutic effects in HCM. To further validate its mechanism of action, we performed a molecular docking analysis of the four characteristic genes with penfluridol. The results showed that the target proteins encoded by these genes were tightly bound to the drug through a variety of intermolecular forces such as hydrogen bonding, hydrophobic forces, and salt bridges, demonstrating high binding efficacy. Visualisation of the optimal binding conformation using PyMOL software further revealed the detailed interaction patterns between the drugs and the target proteins. This finding provides a new research direction and theoretical basis for future drug development against HCM. However, despite the theoretical potential of penfluridol for the treatment of HCM, its off-target effects in cardiac tissues and the safety of its long-term use still need to be further evaluated.

*DUSP1*, *CCND1*, *STAT3*, and *THBS1* play important roles in the pathological process of hypertrophic cardiomyopathy (HCM), regulating myocardial hypertrophy, fibrosis, and cardiac remodelling through a complex signalling network. *DUSP1*, as a member of the MAPK family of phosphatases, inhibits inflammation and oxidative stress by dephosphorylating and inactivating the JNK, p38, and ERK signalling pathways [30,31]. The inhibition of miR-32-5p in a high-glucose environment leads to the sustained activation of MAPK signalling, which in turn promotes cardiac fibroblast proliferation and an enhanced fibrotic phenotype [32], suggesting a protective role in HCM. *CCND1* is a core gene in cell-cycle regulation [33], promoting abnormal cardiomyocyte proliferation and hypertrophy by driving the G1/S transition with CDK4/6 and integrating growth signals such as PI3K/AKT; its aberrant up-regulation also exacerbates fibrosis through the TGF-β/Smad pathway, and the down-regulation of CCND1 may weaken the repair capacity of the myocardium [34]. Moderate activation reduces oxidative stress and inhibits apoptosis, but excessive activation promotes myocardial hypertrophy and fibrosis through the mTOR and NF-κB pathways, and STAT3-deficient mice have reduced myocardial capillaries and interstitial fibrosis, ultimately leading to dilated cardiomyopathy [35,36]. *THBS1*, a multifunctional extracellular matrix protein, promotes myocardial fibrosis by activating TGF-β signalling and reducing microvessel density, and exacerbates myocardial fibrosis by inhibiting angiogenesis, e.g., by blocking VEGF and NO signalling [37]. It reduces microvessel density, exacerbates myocardial ischaemia and oxidative stress, and its expression level is significantly correlated with the degree of fibrosis in HCM patients [38]. Taken together, the four characteristic genes drive the pathological progression of HCM, and a deeper understanding of the functions and interactions of these genes is expected to provide new ideas and approaches for the diagnosis and treatment of HCM.

In summary, this study systematically revealed the centrality of oxidative stress in the pathogenesis of HCM by integrating multi-omics analysis, machine learning algorithms, and single-cell transcriptome data analysis, and successfully identified four characteristic genes (*DUSP1*, *CCND1*, *STAT3*, and *THBS1*) that are closely related to oxidative stress. These genes are not only involved in key pathological processes, such as redox balance, inflammatory response, apoptosis, and myocardial remodelling, but also further exacerbate disease progression by regulating the immune microenvironment and signalling pathway networks. In addition, our drug prediction and molecular docking analyses based on the Cmap database provide important clues for exploring potential therapeutic strategies against HCM. These findings not only deepen the understanding of the molecular mechanisms of HCM but also lay a solid theoretical foundation for the development of precise therapeutic options against oxidative stress-related targets.

However, although this study provides valuable insights into the role of oxidative stress in HCM, it has several limitations. Firstly, the findings are largely based on computational predictions that need to be experimentally validated by detailed in vitro and in vivo studies to confirm their biological relevance. In addition, the dual role of certain genes (e.g., *STAT3*) and the speculative nature of the drug predictions highlight the need for the further investigation of their safety, efficacy, and function in specific settings. Future research should focus on validating these findings in larger patient populations, exploring the functional mechanisms of the identified genes, and conducting preclinical and clinical studies to assess the therapeutic potential of predictive drugs (e.g., pentafluridine). These efforts will help to translate our findings into clinically applicable strategies to target oxidative stress in HCM.

## 5. Conclusions

In this study, we identified relevant targets of oxidative stress in HCM and demonstrated the role of oxidative stress in the pathogenesis of HCM, on the basis of which, potential therapeutic agents against oxidative stress were developed. These findings provide valuable insights for the development of early diagnostic and targeted therapeutic strategies for HCM.

## Figures and Tables

**Table 1:** Primer sequence information.

Genes		Primers (5′–3′)
*ANP (* *ID: 43595* *)*	F	GGCACTTAGCTCCCTCTC
	R	CCCTCAGTTTGCTTTTCA
*β-MHC (* *ID: 140781* *)*	F	TGGATGCAGACCTCTCCC
	R	TGCTTCTTGCCACCCTTG
*DUSP1 (* *ID: 19252* *)*	F	GTTGTTGGATTGTCGCTCCTT
	R	GTTGTTGGATTGTCGCTCCTT
*CCND1 (* *ID: 12443* *)*	F	GCGTACCCTGACACCAATCTC
	R	CTCCTCTTCGCACTTCTGCTC
*STAT3 (* *ID: 20848* *)*	F	CAATACCATTGACCTGCCGAT
	R	GAGCGACTCAAACTGCCCT
*THBS1 (* *ID: 21825* *)*	F	GGGGAGATAACGGTGTGTTTG
	R	CGGGGATCAGGTTGGCATT
*GAPDH (* *ID: 14433* *)*	F	GAGTCAACGGATTTGGTCGT
	R	GACAAGCTTCCCGTTCTCAG

## Data Availability

Dataset available on request from the authors.
